# Enablers and inhibitors of digital startup evolution: a multi-case study of Swedish business incubators

**DOI:** 10.1186/s13731-023-00306-y

**Published:** 2023-05-30

**Authors:** Andrew Page, Jonny Holmström

**Affiliations:** Swedish Center for Digital Innovation, Department of Informatics, Umeå University, Umeå, Sweden

**Keywords:** Digital startups, Digital entrepreneurship, Incubators, Scaling

## Abstract

Global advances in digital technology are facilitating corresponding rises in digital entrepreneurship and its startup manifestation. There are many uncertainties on the road to digital startup evolution, some of which may be successfully navigated with the assistance of business incubators. While these organisations provide valuable guidance and support to the startup community, their efforts are at least partly constrained by the lack of a consistent, coherent roadmap to guide both them and their incubatees. T0 help efforts to develop such a map, this paper seeks to identify factors that influence digital startup evolution within an incubator setting through a multiple-case study focusing on digital startups under the umbrella of three business incubators in the Swedish city Umeå. Sets of enabling and inhibitory factors are identified through literature searches and the case studies. The latter may include inertia and possibly attitudes towards failure. In addition, present the Ideation Dynamics Model as a guide for both incubators and digital startups is proposed.

## Introduction

Modern digital technologies, such as social media, mobile technologies, business analytics, big data acquisition and AI, are opening fascinating innovation opportunities for entrepreneurs (Chalmers et al., 2020; Cohen et al., 2017; Holmstrom, 2022; Holmstrom & Hallgren, 2021). These new digital technologies—regarded as ‘external enablers’ (Davidsson, 2015)—have led to ‘democratization of entrepreneurship’ (Aldrich, 2014) and lowering of the barriers for digital startups by reducing difficulties in the entrepreneurial journey from idea to full-blown firm (Briel et al., 2018). As such, digital entrepreneurship—defined as the practice of pursuing “new venture opportunities presented by new media and internet technologies” (Davidson & Vaast, 2010)—is attracting global attention (Fang et al., 2018; Nambisan, 2017). While this form of entrepreneurship has many similarities to traditional entrepreneurship, a significant difference is that in the former some or all of the key activities are in digital rather than non-digital formats.

For the purposes of this paper, we define a digital startup as a venture that exploits digital technology in its operations, or has a product or service of digital nature. Rapid scaling related to digital startups can be seen as a generative process through which a venture’s user base increases significantly between two points in time (Huang et al., 2017). However, rapid scaling is not eternally generative and requires careful attention by reflective agents to persist (Garud et al., 2010). In the case of digital startups, we argue that such attention involves reflective actors engaging in scaling efforts to increase the user base. While prior research suggests that digital technology plays a crucial role in such scaling, little is known about the mechanisms, whereby digital startups accomplish scaling. Indeed, as digital entrepreneurs adopt new digital technologies to develop novel entrepreneurial actions that accelerate new ventures’ evolution (Huang et al., 2017), the complexity also increases. Hence, many organisations and service providers seek to act as incubators to facilitate entrepreneurial activity, particularly by reducing barriers associated with entrepreneurship through provision of coaching, office space, knowledge transfer and funding (Al-Mubaraki & Busler, 2017; Gonthier & Chirita, 2019; Pettersen et al., 2015; Ratinho et al., 2020; Shankar & Shepherd, 2019; Shepherd & Gruber, 2020). An incubator can be defined in many ways, but according to the Swedish Incubators and Science Parks (SISP) organisation, an incubator is “an entity that offers a dynamic process to developing businesses, people, and companies”. Thus, it assists entrepreneurs with management, financial support and technical competence while facilitating connections to both new environments and a commercial network within which to prosper. It is important for prospective digital entrepreneurs to build, learn and evolve in such incubator contexts. An evolutionary process of particular interest here, pivoting (Ries, 2011), is considered in detail in the following sections.

It has been suggested that the digital and, therefore, relatively flexible nature of today’s startups facilitates more rapid implementation of lessons learnt in such evolutionary processes than in more traditional organisations as “bits are easier to change than atoms” (Huang et al., 2017). Various authors (e.g., Bandera and Thomas, 2018; Kirtley and O’Mahony, 2020) agree that adaptability is highly desirable for entrepreneurs as it allows them to respond more rapidly—for example, by scaling their business—to environmental stimuli. Eesley and Wu (2020) express a more nuanced view, identifying both advantages and disadvantages of flexibility depending on, for instance, the time scale and the advisory networks that an organisation can access and utilise. This echoes recent calls for an increased focus on the role of context in entrepreneurial action (McMullen et al., 2020). However, while endorsing the requirement for startups to react appropriately to environmental stimuli, they do not offer any meaningful guidance regarding the optimal form of responses.

The ‘pivoting’ described by Ries (2011) is undoubtedly important for startups responding to the myriads of environmental stimuli they encounter. Hence, it has received attention from various authors, particularly in the software industry. As noted by Cusumano (2013), for example, without being able to demonstrate the flexibility required to pivot, a startup may struggle to raise essential investment capital. At least 12 types of pivot have been recognized by authors including Bajwa et al. (2016), Bajwa et al.(2017), Ochoa-Zambrano and Garbajosa (2017), Bohn and Kundisch (2018) and Terho et al. (2015). In addition, 14 triggers of startup pivoting (some internal and some external) have been identified by Bajwa et al. (2016), Bajwa et al. (2017) and Comberg et al. (2014).

There are three main types of consequences of pivoting (depending on its success and engagement): scaling, inertia and disintegration/failure. Scaling is defined by Sahay and Walsham (2006) as “the process through which a product or process is taken from one setting and expanded in size and scope within that same setting and/or also incorporated within other settings”. Thus, in the context of this paper, scaling is a substantial increase in operational magnitude by a digital startup to maximise the fiscal benefits of a strategic adjustment, for example, by exploiting the potential of a previously niche product to meet a need in a much larger market. Huang et al. (2017) outline a number of key mechanisms—data-driven operation, instant release and swift transformation—through which the scaling process may occur.

Startup disintegration/failure on the other hand can be viewed as both a positive and negative process. The positive effects associated with failure are emphasised by Gartner and Ingram (2013) and Da Silva et al. (2019). However, while failure may have certain benefits, of course not failing has major inherent advantages. Opinions of failure are highly dependent on the geographical and associated cultural setting. For example, citizens of the USA reportedly have significantly higher propensity to engage in self-employment than European Union (EU) citizens (Bosma and Schjutens, 2011). Since self-employment is associated with a higher risk of failure, it seems likely that at least some of those who pursue it are more comfortable with the associated jeopardy than those who do not. Further differences between the two geographical settings noted by Brenner and Fornahl (2008) include differences in scarcity of funding, bankruptcy consequences, education, culture and fragmentation of member states. All of these factors may influence perceptions of failure. Even within Europe, fear of failure varies significantly between regions and countries according to Bosma and Schjutens (2011), due to factors including its potential negative consequences, underlying unemployment rates and population density. Four strategies to avoid failure specifically in an incubator setting—focusing on the team over the product/service, business model innovation, business model development and business model scalability—are identified by Nair and Blomquist (2018).

Inertia is a state that is generally associated with organisations that are both larger and more mature than startups. Entrepreneurial inertia does not appear to have been widely considered in previous startup research, apart from contributions by Ruef (2006)—who defines entrepreneurial inertia as “the lag time exhibited by organizational founders or investors entering a market niche”—and Gong et al. (2009). Ruef’s definition really refers to a period of inertia, rather than inertia per se, and it can be more simply and accurately defined as a company maintaining its current strategy. This may be due to the company regarding its current course as the most appropriate and having no interest in either testing any of its existing hypotheses or incorporating the results of any such tests into its operational model. Such unwillingness or inability to capitalise upon a pivot—for instance, by scaling up—may also be present in digital startups, but the possibility has not been investigated.

In summary, pivoting of digital startups, its potential consequences (inertia, scaling and failure/disintegration) and influences of entrepreneurial incubators have all received some previous research attention. However, this attention has been patchy, and they have not been addressed in combination. As digital startups operating inside and outside incubators may encounter different environmental stimuli, their enabling and inhibiting factors may differ. Thus, here we address the following research question: *What are the enabling and inhibiting factors in digital startup evolution within an incubator setting?*

Empirical data to assist the effort were acquired in a multi-case study, focused on diverse digital startups within three incubators in the city of Umeå in northern Sweden. Two of the three incubators focus specifically on the life science and creative industries, while the third facilitates organisational development of startups that operate in more varied industries.

This paper is divided into six main sections. Sect. “Literature review” examines extant research concerning pivoting, its triggers, and two of the potential outcomes of pivoting (scaling if pivoting is successful and disintegration/failure if there is no pivoting or it is unsuccessful). Sect. “Research design” examines the methodology applied in the case study and rationales for the methodological choices. Sect. “Results” presents the empirical findings. Sect. “Discussion” discusses and reflects on the relationship between the findings and existing literature, as well as its implications for practices of both incubators and digital startups. Section 6 summarises conclusions and future research opportunities that extend understanding of the focal phenomena. The paper concludes with acknowledgements, a list of references and a brief Appendix.

## Literature review

To gain a thorough understanding of previous work related to the research question, literature relevant to enabling and inhibiting factors in digital startup evolution was reviewed, particularly the relatively sparse literature on these factors in incubator settings. For this purpose, the Google Scholar, Scopus, Association for Computer Machinery Digital Library and Umeå University library’s search tools were used. It would be unnecessarily tedious to list all the search strings used, but they included various combinations of the following terms:

- Startup(s)/Start-up(s)
- Pivot(s)/Pivoting
- Incubator(s)
- Digital Entrepreneurship
- Scaling/Scaling-up
- Disintegration/Failure

Although some authors, such as Comberg et al. (2014), have used the term ‘business model innovation’ synonymously with pivoting, in most published sources the two terms refer to separate phenomena. Papers including this term were, therefore, excluded from the review in an effort to minimise confusion and enhance clarity of thought.

The searches resulted in a primary source catalogue of several hundred papers, online articles, conference proceedings, academic textbooks and theses by doctoral or master level students that either directly addressed focal phenomena or tangentially provided relevant information. Many identified papers consider aspects of pivoting (mostly rooted in the work of Eric Reis), its triggers, scaling, and outcomes of the processes. However, we largely excluded those that do not address these phenomena in the context of a digital startup from further consideration. In addition, if multiple papers covered very similar or identical topics we retained examples that were published in the most highly ranked journals, had the most citations, and/or were by authors with strong publication track records and reputations. This facilitated the streamlining of utilised references to an appropriate level for a work of this magnitude. This facilitated the streamlining of utilised references to an appropriate level for a work of this magnitude.

The literature review identified three potential outcomes (as shown in Table 1) for organizations considering whether or not to pivot: pivoting, disintegration/failure and scaling. This section sequentially summarizes the literature regarding the process of pivoting, its various forms, and diverse factors that trigger it.

**Table 1:** Research streams, definitions and contributory papers

Research Streams	Definition	Supporting papers
Pivoting	Modifying an organisation’s strategy in a manner that does not result in a corresponding change to its vision	Bajwa et al. (2017), Bohn and Kundisch (2018), Comberg et al. (2014), Ochoa-Zambrano and Garbajosa (2017), Terho et al. (2015), Hirvikoski (2017)
Scaling	Maximising the ability of an organisation to achieve rapid growth	Huang et al. (2017), Picken (2017), Brynjolfsson and Saunders (2009), Henfridsson and Bygstad (2013), Sahay and Walsham (2006)
Disintegration/Failure	The inability of an organisation to continue operations in its current form	Bajwa et al. (2016), Bandera and Thomas (2019), Giardino et al. (2014), Unterkalmsteiner et al. (2016), Eisenmann et al. (2013)

### Pivoting

Ries (2011) defines a pivot as “a structured course correction designed to test a new fundamental hypothesis about the product, strategy, and engine of growth”. Applying a slightly different perspective, Kirtley and O’Mahony (2020) describe it as “a change in a firm’s strategy that reorients the firm’s strategic direction through a reallocation or restructuring of activities, resources and attention”. Here, we regard a pivot as a digital startup developing a product or service, testing it in its market, and proceeding in a direction dictated by the outcomes of that process and associated lessons.

Bajwa et al. (2017) have shown that pivots often result from analysis of corroborated customer feedback regarding a particular hypothesis or product/service. Such feedback forms a central component of Ries’s (2011) Lean Startup approach, which involves the development and testing of a premise and subsequent incorporation of the results into a startup’s decision-making processes. Other authors claim that pivoting may result from either efforts to facilitate the matching of a product to a market need or failure to achieve that objective (Giardino et al., 2014; Ochoa-Zambrano & Garbajosa, 2017).

There are divergent views concerning the magnitude of strategic change required for a pivot. While Bandera and Thomas (2018) believe that the scale of change associated with pivots can be both significant and small, others such as Ries (2011) and Blank (2007) regard them as major adjustments in the journey of a startup.

The experimental work associated with pivoting can be regarded as the antithesis of what Crilly (2017) refers to as fixation; the idea of remaining loyal to a group of beliefs, thereby reducing or removing the potential for absorbance of learnings and occurrence of pivoting. Similarly, Cusumano (2013) suggests that successful pivots require a significant degree of organisational flexibility.

### Scaling

As already mentioned, Huang et al. (2017) describe scaling as a generative process through which a venture’s user base increases significantly between two points in time. Sahay and Walsham (2006) offer the following, more expansive definition; “the process through which a product or process is taken from one setting and expanded in size and scope within that same setting or/and also incorporated within other settings”.

Scaling often follows, and is enabled by, a successful pivot. Brynjolfsson and Saunders (2009), Henfridsson and Bygstad (2013), and Yoo et al (2010) have shown that digital scaling differs from, and can be much faster than, its traditional counterpart due to the ability to utilise and build on existing digital assets. Henfridsson and Bygstad (2013) also describe a scaling mechanism through which startups can expand their reach via their digital infrastructure. According to this mechanism, organisations can expand the reach of their digital infrastructure and reach previously untapped customers by offering enticements to potential partners—who then become actual partners. The process is, therefore, self-perpetuating. It also creates a level of innovation space by providing the infrastructural augmentation required to enhance reach.

Sahay and Walsham (2006) emphasise that while numbers and size are important elements of scaling, the phenomenon involves far more. The dynamics that facilitate the spread, enhancement, scoping and enlargement of the heterogenous networks associated with (or through) the technology must also be considered. They also demonstrate that an organisation’s ability to scale successfully may depend on such factors as technology, people and processes, as well as the contextual setting.

Picken (2017) outlines several organisational requirements and actions—associated with what he terms rapid scaling—that a startup must have and execute for success. Compared to earlier points in a startup’s journey he emphasises the need to substantially enlarge the company’s resource base while utilising processes and alliances to expand the venture, in accordance with a corroborated commercial hypothesis and sustainable plan. He also identifies the internal changes that must occur—relative to the startup’s earlier form—for successful scaling; specifically, modifications to such staples as configuration, regulation and process. Finally, he defines the objective of scaling as “rapid growth to achieve competitive scale and establish sustainable market leadership”. In addition, Huang et al. (2017) identify three distinct mechanisms, whereby digital innovation can result in rapid scaling: data-driven operation, instant release and swift transformation. Data-driven operation refers to the process of detecting and responding to potential opportunities—and corresponding risks—through high-volume data analysis. Such analysis can facilitate a startup’s ability to frame, hedge and monitor potential upsides and downsides, before and during rapid scaling.

The concept of data-driven operation has the following three distinct components.

- *Data profiling* The utilisation of user data by a startup to distinguish and consider potential untapped areas of opportunity (e.g., clusters of new users).
- *User hedging* The use of as many and as diverse data sources as possible to generate a balanced risk profile for each innovative activity a startup considers in its developmental plans.
- *Fine-grained monitoring* The scrutiny, at as granular a level as possible, of a startup’s user metrics to identify areas of operational concern.

### Failure/disintegration

In the context of this paper, as discussed by Ries (2011), failure/disintegration is a possible outcome of an organisation failing to pivot, or pivoting in the wrong direction. Many organisations celebrate failure, primarily for the resulting learnings that may be absorbed and repurposed. As Thomas Edison reportedly said, according to Da Silva et al. (2019), although numerous variants have been quoted, “I have learned fifty thousand ways it cannot be done and therefore I am fifty thousand times nearer the final successful experiment”. Gartner and Ingram (2013) claim that entrepreneurs tend to agree with Edison and view failure positively. Clearly, however, the failure of an entire startup—with its corresponding implications for employment, personal financial status, reputation and future endeavours—is not a desirable outcome, whatever wisdom may be acquired from the event.

Startups by their very nature—which Cantamessa et al. (2018) define as high-risk and high-reward—are prone to relatively high failure rates. Estimates of this rate vary, but 90% is an oft-quoted figure (Marmer et al., 2012).

Failure may refer to an organisation or solely to a particular product or service that it offers. Blank (2007) proposes that the principal reason for digital startups’ failure is not the technology that underpins their product offering but rather a lack of customers and associated inability to generate the user data required to facilitate the development of both the product and targeted market. Blank (2013) further proposes that startup failure rates can be reduced by applying Eric Ries’s Lean Startup methodology. More specifically, failure is an outcome that startups can minimise either through constant customer feedback-mediated iteration and adaptation of their concept or pivoting away to a concept with more potential.

Ries (2011) debunks the mythical links between startup success and creativity, work intensity and determination. He argues that constraints associated with traditional management techniques and philosophies—such as fear of failure, lack of flexibility and short-termism—are incompatible with startups and thus liable to exacerbate the underlying risk of failure. He also maintains that a new discipline of entrepreneurial management is required to overcome this deficiency. In a similar vein, Crowne (2002) identifies and discusses several specific causes of software startup failure, including inexperienced developers, lack of product owners and dearth of cohesion between the organisational strategy and components of the product offering. However, according to Nair and Blomquist (2018), digital startups operating within an incubator setting—the objects of this work—are less prone to failure than those outside, due to application of the following strategies.

- *Focusing on the team around a scalable idea* Startups clearly require an idea of some sort to bring to market. However, incubators tend to focus more on the qualities of the startup team and whether they possess the potential to scale up the idea to a commercially viable level, rather than the perceived innovativeness and superiority of the actual product and/or service.
- *Business model validation* Incubators are very aware that flaws in startups’ business models are the commonest causes of failure. Thus, they encourage resident startups to pressure-test their business model by repeatedly, and as soon as possible in their lifecycle, taking a minimum viable product to potential customers, obtaining feedback and incorporating that feedback.
- *Business model development* As a startup progresses through its incubator journey, it must demonstrate proof of concept at various points to maintain its funding streams. Incubators can provide startups with the myriad resources they require to reach these points, the required support to pivot to a new idea if the concept cannot be proven, and facilitated access to finance streams to support them, while they undertake these activities.
- *Business model scalability* Most incubators want their startup residents in and out of their premises as rapidly as possible. This desire can be met through either the ability or clear lack of ability of a business model to scale, and incubators can facilitate construction of a scalable and feasible commercial model through coaching, mentoring, and access to networks.

## Research design

The design of the empirical research this paper is based upon is summarised in this section, which sequentially describes (with justification from the literature) the research approach, case study and sampling method, data collection, data analysis, and ethical issues.

### Research approach

The focal phenomena in this study are rooted in complex processes and socio-technological interactions that are not inherently amenable to quantitative analysis, so a qualitative, interpretivist approach was adopted, seeking to unmask interviewed agents’ views of reality (Holmström & Sawyer, 2011). Specifically we sought to gather thoughts on factors that have enabled and inhibited digital startups’ evolution. This is clearly aligned more with interpretive than either positivist or critical schools of thought, as defined by various authors (e.g., Dubé & Paré, 2003; Myers, 2013).

The focal objects were resident start-ups in three incubators, so multiple-case methodology was applied to explore the startups’ evolution and navigation of their ecosystem, through collection of rich information from multiple sources, as recommended by Hannah and Eisenhardt (2018). The approach is also consistent with advice by Mills et al. (2010) to select “several instrumental bounded cases… to develop a more in-depth understanding of the phenomena than a single case can provide”.

Multiple-case study was selected as it seemed the most appropriate methodology for examining factors influencing digital startup evolution in an incubator setting in the light of more general extant studies. Various authors, such as Hannah and Eisenhardt (2018) and Eisenhardt et al. (2016), have demonstrated the suitability of multiple-case studies for elucidating processes such as those considered here, and others have provided clear guidance for rigorous application of interpretivist approaches in IS research (Dubé & Paré, 2003). Moreover, according to Myers (2013), case studies are appropriate for modern-day and actual scenarios, where the researcher does not seek to—and cannot—control any aspect of the research process. He also notes their suitability for studies focusing on how and why something occurs in complex processes.

It should be noted that case studies have various potential limitations and flaws, as listed by Dubé and Paré (2003), many of which are associated with poor execution. For example, as noted by Myers (2013), understanding which case study data to retain and which to discard can be challenging, especially for researchers with little experience, as everything may seem relevant.

The advantages, disadvantages and potential suitability of a number of other research methods—Grounded Theory, Action Research and Ethnography—were considered, with consultation of discussion and recommendations by Myers (2013) and Yin (2018), before making the final methodological choice.

### Case study elucidation and sampling

The focal cases were 13 startups being nurtured by three business incubators in Umeå, Sweden: the Umeå Biotech Incubator (UBI), BIC Factory (BICF) and eXpression Umeå (EXU). These organisations have similar geographic locations and ownership structures, but focus on very different industries. Typically, startups stay within the incubators for 2 years, but this period can be extended if both parties agree that it would be beneficial. Startups in multiple incubators rather than one were selected to broaden the environmental and contextual relevance of the acquired data. Managers of each incubator were contacted by the second author and facilitated contact with key stakeholders of the startups.

UBI positions itself as a biotech incubator operating within the northern Swedish life science industry. It mentors successful applicants through a multi-stage development process—including concept verification, funding and growth—designed to ensure they achieve commercial success upon graduation. eXU focuses on a diverse range of creative and cultural industries, aiming to advance partner organisations through provision of four distinct developmental programs—Express, Creative, Future Retail and Design—implementation of which is enhanced by the innovative and stimulating environment within which day-to-day activities take place. BICF is a general business incubator specifically focused on young entrepreneurs that provides 2 years of leased working space, commercial mentoring, and the possibility for startups to construct and benefit from a robust business network with a range of potentially useful partners.

Time and manpower limitations prohibited use of representative (probabilistic) sampling methods (Etikan et al., 2015) to identify enabling and inhibiting factors in digital startup evolution. After consideration of the pros and cons of possible non-probabilistic techniques, as described by Bernard (2017), purposive sampling (also known as judgement sampling) was selected. This refers to choosing informants with relevant characteristics (Tongco, 2007), here positions as senior managers and or/owners of startups that have pivoted and reside in an incubator. Purposive sampling is regarded as suitable for various types of case studies, and sometimes crucial for sampling sparse populations (Bernard, 2017), such as startups in incubators in a single small city like Umeå (population 120,000 people).

Alternative nonprobability sampling techniques that were considered—and are further discussed by Bernard (2017)—included quota sampling, convenience (or haphazard) sampling, and chain referral (snowball and respondent-driven) sampling. Quota sampling was not suitable as we were not seeking to obtain specific proportions of populations. Convenience sampling (interviewing anyone who can be easily accessed) seemed unsuitable due to the highly specific research question, and chain referral (relying on disclosure and subsequent referrals by interviewees of potentially suitable subjects) seemed to leave too much to chance in a time-constrained project.

### Data collection

The primary data sources were 13 interviews—which are generally key elements of such case study research (Myers, 2013)—with senior managers or owners of startups hosted by the incubators. To allow interviewer flexibility and creativity together with free expression of the interviewee the interviews were semi-structured (Diefenbach, 2009), following a script divided into both individual questions and three sections, as recommended by Miles and Huberman (1994).

The interviews were conducted via Zoom, and audio-visually recorded for thematic analysis. Several interviewees were aware of and specifically referred to the work of some authors whose publications underpin this paper. However, in an effort to minimise these authors’ influence on their responses we did not proactively raise such matters with any participant.

The first part of the interview guide focused on the background of the interviewee and other members of their startups’ senior management teams. They were specifically asked if their startup had ever experienced rapid scaling (after sharing definitions of the phenomenon to facilitate comprehension) or failure. The second part concentrated on multiple pivoting-related topics. At the start of the second section of each interview, definitions of pivoting were shared with the participants to maximise understanding of the term. The concluding section focused on their experiences in the incubator, with an emphasis on both pivoting and areas of their business that had been most strongly influenced by their respective incubator partners. The interviews varied in length from 40 to 75 min (mean, 49 min). Information regarding the participants and their interviews is provided in Table 2. In efforts to protect their anonymity, bearing in mind the relatively small size of the sample, the sectors in which their organisations operate have not been identified, but they included CE marking, creative, retail, internet-service provision, life sciences, media production, nutrition and software industries.

**Table 2:** Interview details

Participants	Interview Duration (min/sec)	Incubator
Startup A	46.19	BICF
Startup B	49.51	UBI
Startup C	45.57	BICF
Startup D	49.57	eXU
Startup E	49.24	BICF
Startup F	48.37	BICF
Startup G	50.20	UBI
Startup H	75.48	UBI
Startup I	40.36	BICF
Startup J	58.24	eXU
Startup K	39.44	BICF
Startup L	48.24	eXU
Startup M	40.05	UBI

Since, as already described, the participants were not chosen on a random basis and this was an interpretive study, there was clear potential researcher bias. Managing bias is an important factor for researchers to consider, as it can severely undermine the validity of research (Collier & Mahoney, 1996; Mehra, 2002). Notably, the relative intimacy of the relationship between researcher and subject, together with frequency of contact, in case studies can result in interviewees being less than frank and honest in responses, because they want to protect themselves or their organisation (Holmström & Sawyer, 2011). In this study the potential for bias was minimised by both the brevity and scarcity of the interactions between the interviewer (first author) and interviewees, and strict pledges of confidentiality with minimization of possibilities of identifying them or their organisations in any resulting publication.

### Data analysis

Thematic analysis, according to Braun and Clarke (2012), is “a method for systematically identifying, organizing and offering insight into patterns of meaning (themes) across a data set”. This was regarded as the most appropriate approach for identifying the focal enabling and inhibiting factors due to the requirement to identify common themes, the method’s flexibility and simplicity (Braun & Clarke, 2006), and its suitability for analysing contents of in-depth interviews (Guest et al., 2012).

Other data analysis methods—described by Bernard (2017) and Leech and Onwuegbuzie (2008)—that were briefly considered from consideration included hermeneutic or semiotic approaches and narrative analysis, but they were subsequently excluded for the following reasons. Hermeneutic approaches are most useful for enhancing understanding of people rather than processes of commercial entities, semiotic analysis for interpreting signs and symbols (which were not considered here), and narrative analysis for capturing stories rather than key themes or commonalities and differences between cases.

**Fig. 1:**
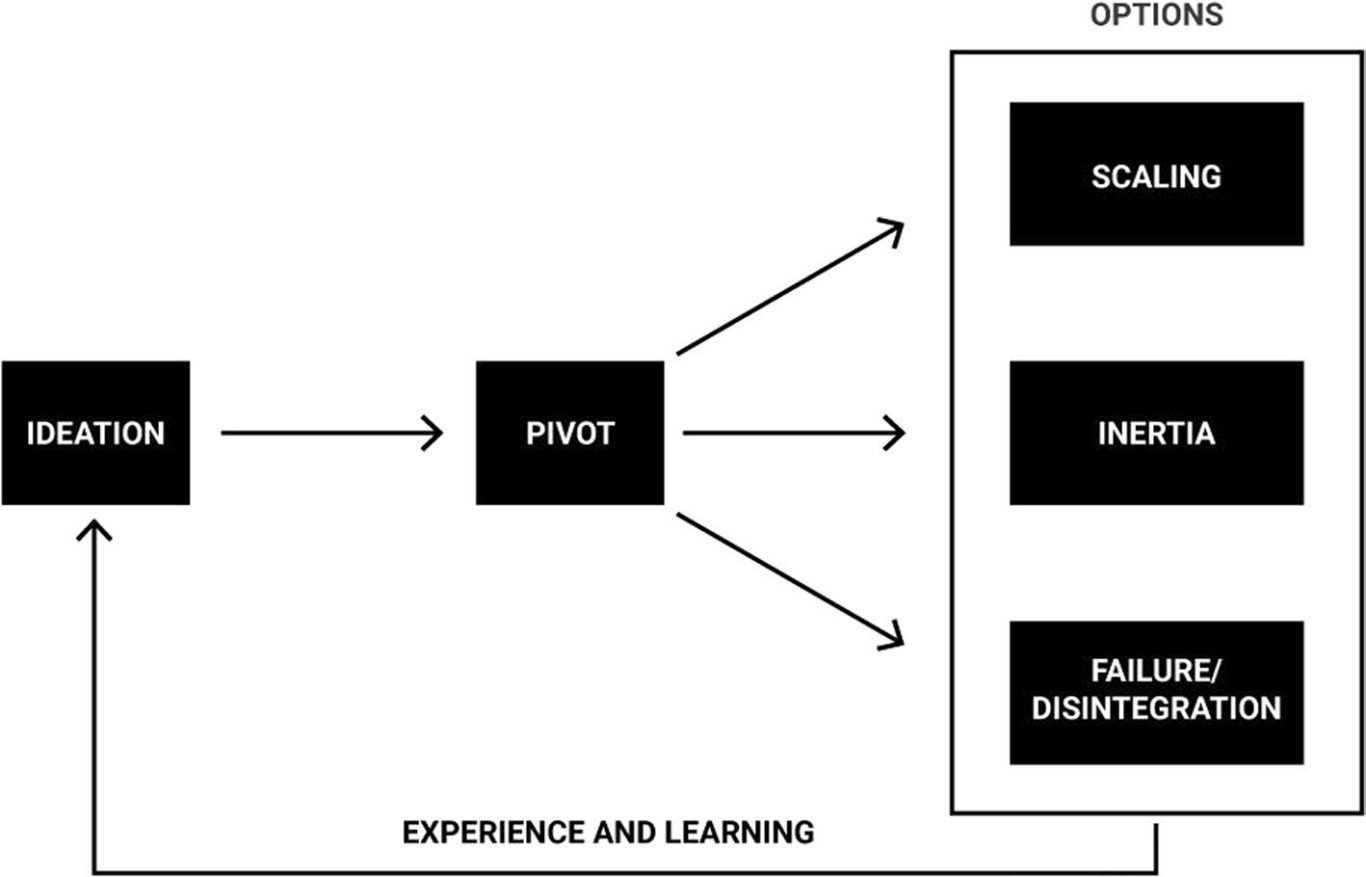
Schematic representation of the Ideation Dynamics Model

The primary objective of the analysis was to extract relevant themes from the material. During an initial review, a number of preliminary codes were manually identified and considered, then the ATLAS.ti analytical software package was used for more in-depth review. Four themes were identified—incubator influence, pivoting, scaling, and failure/disintegration—which are strongly related to each other, as schematically illustrated in Fig. 1. An additional objective was to connect, through iterative coding, key findings from the interviews with content in corresponding research streams identified in the literature review. In this process a number of embryonic ideas were recognised then compared to, and contrasted, with previous findings.

### Ethical issues

In efforts to avoid the potential negative outcomes of failing to consider ethical factors in research (Bernard, 2017), we endeavoured to consider any potential issues that might arise before, during and after the interviews (Ritchie et al., 2013). All potential study participants (14 in total) were contacted twice by email to request an interview. If no response was received, contact was discontinued, but only one potential interviewee failed to respond to two invitations. The emails clearly communicated the study’s purpose and its rationale, together with an explanation of the information we were seeking to obtain from the interviews. The expected duration of the interview was also disclosed. Once a time and date were agreed, a Zoom invite and calendar appointment were sent to the interviewees.

At the beginning of each interview, the purpose and rationale of the research were communicated again and participants were asked if they had any questions. Consent to record the discussions and utilise any data generated in an anonymised manner was sought and obtained. The anticipated duration of the call was also mentioned again. Every effort was made to make interviewees feel at ease. Interruptions were kept to a minimum and not initiated by the researchers unless it became clear that the conversation was veering completely off-topic or a question had been misinterpreted. At the conclusion of the calls, participants were asked if they had any additional questions for the interviewer or any final thoughts on the topic they wished to express.

With such a small sample size, complete confidentiality cannot be absolutely guaranteed, but the pledge made to maintain confidentiality and respect interviewees’ privacy has been protected as far as possible during reporting of the results. Several participants requested a copy of any publication using material recorded in the interviews, and we will ensure that they all do so.

## Results

This section of the paper presents empirical findings of the research. Five distinct themes related to the research question emerged from the interview content coding, which have robust interdependent relationships. These five—incubator influence, pivoting triggers, scaling, failure/disintegration and inertia—and summaries of the results concerning them are presented below.

### Incubator influence

This section summarises opinions expressed by the interviewees regarding the associated incubators’ influence on their digital start-up’s operational dynamics. All of them were, to a greater or lesser extent, satisfied with at least some aspects of their incubator experiences, and appeared to understand the inherent value of their association with these umbrella organisations, particularly the access to a wide range of external (and complimentary) services it enabled. They mentioned such services as accounting, financing, legal (contract and IP), marketing and regulatory advice. They generally regarded internal coaching as having equivalent value to the external services, including advice and input on such matters as brainstorming, investor management, IPO preparation, motivation, presentation skills, price-setting, prioritisation, productivity optimisation and sales training. Finally, the public exposure, networking opportunities (internal and external) the incubators enabled, as well as the financial benefits like subsidised office space, were frequently mentioned. Participant G summarized thoughts on the incubators’ importance by stating “without them, from the beginning, I don’t think we’d have managed to take this [startup G] to where we are now”. Similarly, Participant C stated that “I’ve been very happy with the support I’ve received. The coaching has made a real difference to my company.”

However, four of the interviewees also expressed a degree of dissatisfaction with their incubator mentorship related to a lack of sufficiently specific expertise and skill-sets. While they were grateful for the wide range of coaching available, a recurrent theme raised by a small but vocal group was the lack of relevant experience possessed by some managers and/or coaches in the incubators. There was an underlying belief that no-one with general experience or expertise rooted in a different industry would be able to provide the specialised counsel they required. Participant D, for instance, felt that “The most valuable form of mentorship you can get is from people who have done what you’re trying to do and I kind of feel that none are available”. Similarly, Participants C and H who, respectively, opined that “They are always people, who maybe in a best case were running a company maybe 15 or 20 years ago, but maybe don’t have much experience of today’s environment” and “The ones that have the experience, they don’t work for a governmental funded agency in any country”.

All 13 interviewees reported a lack of mentorship related to pivoting and pivot triggers. While incubator coaches reportedly offered occasional aid with strategy development and/or challenged startups’ strategies, none of the interviewees indicated that an incubator manager and/or coach had suggested execution of a pivot.

To summarize information gathered regarding incubator influence, the incubators seem to focus on assisting the organisations both tactically and operationally. Their efforts and influence in this respect are both considerable and appreciated by the startups. However, their influence does not extend to pivoting. Representatives of the incubators and their incubatees discuss strategy, but not the adjustments and refinements associated with pivoting. Responses corroborating this limitation included “I wouldn’t say they’ve done that, they’ve left that to me” (Participant A), “They haven’t given any advice like that, the advice we usually get is to focus more” (Participant E), and “I don’t think so, no, it’s been more about pushing me forward than rethinking” (Startup F).

### Pivoting

This section records the participants’ recollections on both the pivoting activities they have undertaken and opinions regarding identification of the root causes that led to changes in organisational strategy. All of the participants, except Participant I, claimed that their startups had executed at least one change of strategic direction that they felt qualified as a pivot. However, some of these changes would not qualify as pivots under any of the definitions in the extant literature. Thus, these events are not included in the results section.

The changes offered as examples of pivots varied widely. Examples of activities specifically related to refining the strategy of a functional area of the business included platform change, product divestiture, new market entry, marketing digitalisation, business model innovation, expansion or modification of a service or product, replacement of key partner(s), director removal, pricing strategy refinement, new customer focus, sales channel addition or elimination, production insourcing and substituting consultants for employees.

In one major reported pivot, Startup A completely reconfigured its customer-facing platform to offer clients a greater level of portal personalisation than their previous infrastructure could provide at a moderate cost. Participant A described the implementation process as “very successful, beyond our expectations”.

Startup E’s pivot radically differed, and focused on the people who undertook the development work that their organisation relied upon. They moved away from partnering with an IT consultancy based on Sweden that assigned their work to a wide and inconsistent range of developers and instead partnered with a Vietnam-based consultancy that recruits and manages staff—selected by the startup—specifically and exclusively for their projects. They believed this arrangement offered them a number of advantages and Participant E explained “We got developers that are really good, they feel they work for us. In their minds they’re our employees. That’s what we want to achieve, we don’t want them to be employees for a consultancy”.

In contrast, startup I had been on the point of pivoting several times, and believed that their original (and current) course remained the most efficacious for the organisation’s financial success according to its interviewed representative: “We’ve been on the verge [of pivoting] many times, but actually we’ve strongly believed in our main plan”.

In a final example, startup L executed a pivot that involved a change in their positioning within their industry’s supply chain continuum, shifting their entire business model from being a media developer whose work was distributed by others to a distributor of content developed by other organisations.

#### Pivoting triggers

The factor(s) identified by the digital startups as having triggered the pivots were also extremely diverse. They included: lack of sales, customer feedback, customer needs, the coronavirus pandemic, excessive costs, supply chain disruption, loss of vendor oversight, creative inertia, regulatory legislation and lack of relevant internal skillsets.

Customer feedback was a recurrent element of this theme during the interviews, with three organisations—startups B, C and G—all presenting their customers with either a minimum viable product (MVP) version of their product or a detailed explanation of it to gather feedback.

Financial considerations, including cost reductions or revenue increases, were cited by several of the participants as key parts of the rationale underpinning their pivots. For example, in addition to the pivot mentioned above, startup L moved distribution channels from physical settings to an exclusively online presence in an effort to reduce their cost base and increase profitability.

For Startup D, according to its interviewed representative, the presence of much of their vendor partner network in a myriad of developing countries fostered communication and fulfilment issues that necessitated constant change in their supply chain strategy.

Startup H reportedly realised that they had niched their innovative patient-focused technology and that its utility could be expanded across a far wider population than previously envisioned. The solution, according to Participant H, “was applicable in pretty much any context within healthcare; it opened up possibilities that we hadn’t really thought about.”

Changes in European Union (EU) regulations resulted in enforced changes—and consequently significantly greater costs—for the product development strategy of both startups G and M. As Participant G explained, “Unfortunately it’s something that’s completely beyond our control but it has demanded a complete rethinking of our strategy”.

The colossal impact of the novel coronavirus on socio-economic well-being globally has undoubtedly triggered some very recent and swift pivoting by a number of Umeå-based startups. Some participants acknowledged that this had disrupted their business, but also created opportunities. Startup C, for instance, had transformed its key marketing tactics from a print-based to a multi-faceted and integrated digital campaign. However, their ability to attend key customer meetings and conferences had also been curtailed. While they appreciated the cost reduction opportunities offered by their transition to digital marketing, they felt that this benefit was overshadowed by the inability to interact directly with customers.

Other participants acknowledged the fragility of startup organisations that were experiencing financial difficulty after just a few weeks of disruption. For example, Participant B commented that “it makes you wonder how these companies are being run if they’re becoming insolvent after only a few weeks”. It should, of course, be noted that most of these interviews took place in April 2020, before the enormity of the COVID crisis had been fully revealed to the world.

In summary, most of the digital startups seemed to have pivoted at some point, but some claimed pivots did not meet standard criteria. The breadth of both the pivot types and their triggers were diverse and associated with equally diverse aspects of the companies’ environments.

### Scaling

None of the participants’ digital startups except B had undergone rapid scaling during their organisational lifespan. A number of the organisations felt that it was simply too early in their lifecycle to do so—or the period of time after pivoting was too short—for any scaling activities to have taken place. However, this was by no means the case for all of them. In other cases, scaling was not practical because of simple manpower issues. These were major constraints (for example) for startups whose chief marketable commodity was time and only had one employee/owner whose hours could be billed and had no plans to increase their headcount.

One of the reported primary motives for Startup A’s decision (discussed above) to pivot to a proprietary platform that facilitated client personalisation was the potential ability to scale their business in the future. They contrasted their previous business model of creating stand-alone educational courses, which were owned by their clients, with current activities centred around creating proprietary content that can be branded for their client’s needs. Specifically, Participant A stated that “Our main focus will be to have ownership of the courses, and that’s because of the scalability”.

Inherent characteristics of Startup D’s business model—based on facilitation of access to physical products by digital means—would have reportedly constrained their ability to successfully scale. However, in this case, the current ability of the startup’s supply chain to rapidly increase manufacturing output would be problematic, causing Participant D to assert “You are kind of limited by what your production partners can produce”.

At the time of the interviews, Startup E preferred to avoid rapid scaling, due to a perceived lack of the required internal management expertise. Participant E explained, “That’s what we’d like to achieve, but we wouldn’t know where to start with something like that.”

In contrast, startup B had enjoyed exponential growth in sales during the preceding 12 months. The company attributed this successful outcome primarily to their ability to gather and publish long-term data showing potential clients that the operation of one of their proprietary solutions—which at the time only existed as an MVP—could scale up in a manner that had not previously been recognised or understood. Participant B also emphasised the internal challenges associated with publishing information-based solely on data generated from an MVP rather than a final product; specifically, from the more technically minded team members who believed such actions to be a significant error of judgement.

A number of the digital startups had not scaled in any sense of the term, for several reasons. Some lacked the ambition, necessary partnerships, and/or skills and capabilities, while others felt that the right time in their organisation’s lifecycle for such an exercise had not yet come.

### Failure/disintegration

This section focuses on the concept of digital startup failure/disintegration as discussed with the interviewees.

None of the interviewees admitted any failure of their organisations, either in their current form or in a previous iteration. Moreover, none admitted any failures in previous or concomitant commercial activities. Far more than any other, this part of the interview provoked the least amount of discussion and willingness to engage. A highly significant element of this topic was, of course, their definition of failure/disintegration. Some, such as Participant E, seemed to associate the concept with total financial collapse of the enterprise, with failure to continue trading through inability to pay debts. However, others (such as Participant D) felt that failure could refer simply to arriving at a point in the organisation’s journey, where it had become clear that their idea was not going to enjoy widespread commercial success, possibly due to the company’s relatively young age. Finally, some acknowledged that certain initiatives within their digital company had failed, but had not led to failure of the entire organisation. For example, Participant K described having had “failed projects but nothing more”. Similarly, Participant H stated that “There’s stuff we did that we look at now and think, how could we…”.

Some respondents were eager to emphasise the previous successes that either they or colleagues had enjoyed when failure was raised. For example, Participants M and I, respectively, stated that “My cofounder’s been in two successful startups, so I guess that’s a good thing” and “I’ve been involved in a number of successful companies before this one”.

Furthermore, some of the interviewees claimed to have been aware, or even in close proximity, of digital startups that had failed. A member of Participant L’s team had experience of actually supervising such companies through his previous employment by an incubator—in addition to managing his own company—and commented that “I worked in an incubator where we had success stories and less successful stories”.

Participant L also emphasised that he and his colleagues believed that the frequency of failure was relatively low in the creative and cultural industries due to the relatively low levels of overheads and debt incurred by many digital startups in them. He also noted that external investments accepted by startup entities in these industries were often in the form of non-repayable grants: “If they get funds it usually comes as soft money rather than loans or venture capital, so there’s a less failing culture in creative businesses”.

Finally, a partial contradiction to the views expressed earlier in this section emerged when the interviewer proposed that failure should be seen—as it is in other parts of the world—as at least partially positive due to its potential learning benefits. Most interviewees agreed that commercial failure could indeed be beneficial. However, whether this was a true reflection of their views or simply the adoption of a nonconfrontational position is difficult to ascertain with any certainty.

Failure/disintegration appeared to have been surprisingly rare or even non-existent in the interviewees’ startups. Although different parties assigned different meanings to the term, it was not an outcome that triggered a great deal of engagement and in some cases led to (brief) discussions concerning either digital startup successes or failures experienced by other parties. However, mere lack of debt may have been the main criterion for participants’ conception of failure, rather than broader perspectives.

### Inertia

Inertia was a prominent theme of participants’ comments. One of the most common, interesting and unexpected elements of this theme was that despite claims that almost every organisation had executed a pivot, only one participant acknowledged a subsequent (or even previous) experience of scaling or failure/disintegration. The default action appeared to be to maintain a growth/lack of growth trajectory similar to the pre-pivot trajectory. This apparent lack of dynamism was perhaps best encapsulated by Participant E stating that pursuing anything other than inertia would “be a problem for us right now” due to current lack of ability to scale.

One rationale for inertia that emerged during the interviews revolved around perceived difficulties associated with funding an escape from such a state. Some of the startups were entirely self-funded by the owners or, as in the case of startup F, close friends and family. A view expressed by participants associated with some of these startups, such as C and E, was that a prudent approach was essential due to the currently suboptimal global economic environment and their focus should be on survival, albeit in a state of relative inertia. Participant C declared “Now, with the coronavirus, I’ve had to reduce my costs as much as possible to survive”, while E asserted that “until coronavirus passes and we can seek investments to strengthen our financial position, those plans [to expand the company’s offer to customers outside the EU] are on hold”. In contrast, others—G and M for instance—relied on such sources as ‘soft’ money, venture capital and additional investments from their board of directors and current shareholders to maintain current levels of activity. Representatives of these startups expressed that the mentioned environmental factors would make adequately funding an escape far more challenging than normal. Participants G and M were acutely aware that they might need to cede control of their enterprises to finance an escape from inertia, and Participant M stated ‘that’s not a decision that I’m in a big hurry to take”.

Startup F was reportedly reluctant to compromise creative principles and aspirational branding by undertaking endeavours felt to conflict with the work they most wanted to do. In fact, the company had actively declined work that they felt interfered with these goals, and expressed reluctance to interfere with what they regarded as an acceptable work–life balance and recognised that for the company to achieve its full potential it would be necessary for this equilibrium to be disrupted. Participant F averred, “If it was all about the growth and money, I wouldn’t be here in Umeå, I’d be in Stockholm”.

Finally, it can be argued that the findings regarding incubator influence (or lack thereof) are also relevant to inertia. While no participant specifically cited a link between their startup’s lack of scaling or failure and behaviour of their respective incubators, it seems plausible that the dearth of guidance regarding pivoting could be connected to the rarity of these post-pivoting dynamics.

In summary, inertia emerged as a prominent theme in the interviews. It appears to occur for diverse reasons, including factors associated with finance, creative principles, the novel coronavirus and lack of motivation. It is also strongly related to and influenced by the presence of factors linked to the other themes that emerged in the interviews.

## Discussion

As noted by Islam et al. (2017), “digital technologies offer multiple opportunities for firms but they also involve many challenges”. Some previous studies have examined factors that facilitate or impede efforts to exploit the opportunities and overcome the challenges in digital startup evolution. However, an examination of the factors that facilitate or impede efforts to exploit the opportunities and overcome the challenges in digital startup evolution in the context of incubator settings have received very little attention. Acquisition of such knowledge could substantially enhance the long-term prospects for startups in such settings, with associated benefits for their geographical settings, through improvements in tax receipts, employment, investment, collaborations, and various other valuable societal benefits. Thus, we sought to address a significant research gap by identifying the enabling and inhibiting factors in digital startup evolution in an incubator setting.

Since the study is rooted in digital entrepreneurship theory, it is important to recognize the key differences between traditional entrepreneurship and its digital successor. Clearly, as noted by Nambisan (2017), Henfridsson and Yoo (2014) and Fang et al. (2018), while they have substantial similarities, there are also substantial differences. The latter include differences in entrepreneurial practices, greater fluidity and/or fewer constraints and stronger entrepreneurial agency with less predefinition and more distribution in digital versions.

The rest of this part of the paper describes contributions of the findings to digital entrepreneurship research, compares and contrasts them with previous findings, suggests avenues for future research and briefly discusses the study’s limitations. Sect. “The Ideation Dynamics Model (IDM)” discusses in greater detail the Ideation Dynamics Model illustrated in Fig. 1. Sects. “Inertia” to “Failure/Disintegration” successively address inertia, pivoting and failure/disintegration. Finally, Sect. “Implications for practice” considers implications of the findings for practice of both incubators and digital startups.

### The ideation dynamics model (IDM)

The IDM essentially refers to a series of paths that a digital startup can follow from an initial concept. Two paths—inertia and failure/disintegration—do not lead to long-term success and inhibit the evolution of digital entrepreneurship. Scaling, in contrast, is an enabling process that is essential for a digital startup to achieve its full potential. The schematic diagram in Fig. 1 is intended to communicate the concept that a future product and/or service—represented by the idea box—should be tested by a digital startup with its target audience to ascertain whether a pivot is required. This helps to ensure that the idea, when it takes the form of a product or service, meets needs in its intended market. Following a pivot, the results suggest, in combination with previous findings, that there are three options for the organisation: scaling, inertia or failure/disintegration. Scaling is clearly the most favourable outcome and failure/disintegration the least desirable, while inertia is in an intermediate position.

As illustrated in Fig. 1, whichever of the three paths is taken, the process should exhibit characteristics of a continuous closed loop system. Therefore, whether the idea is subject to scaling, inertia or failure/disintegration, a digital startup should always be searching for its next concept to evaluate and guide through the IDM. The experience and lessons that will undoubtedly be obtained from each product development lifecycle will feed back—and hopefully offer considerable potential benefits—into the succeeding development cycle.

While each of the three outcomes—scaling, inertia and failure/disintegration—have been examined, either as stand-alone phenomena or in different configurations to the model presented here, no similar model has been published in the reviewed digital entrepreneurship literature. The IDM may make an important contribution to the existing Lean Startup model of Ries (2011) by introducing an enhanced level of sophistication and complexity, and thus potentially facilitating more accurate outcome modelling. Awareness, knowledge and understanding of this simple basic model may provide important developmental support for budding entrepreneurs and guidance for incubator managers and coaches. This support may—even if simply by raising awareness of these phenomena—help to maximise scaling while minimising inertia and failure/disintegration, thereby promoting the previously mentioned socio-economic benefits.

### Inertia

This section discusses the third outcome of digital entrepreneurship identified in the case studies and included in the IDM: inertia. There appears to have been very little previous research on inertia in the digital startup community, and Ries (2011) only recognized two outcomes for digital startups: scaling or failure. In contrast to this neglect, our results clearly suggest that it may be a common phenomenon in at least some digital startups.

Reasons for inertia in digital startups may include links between organisational inertia and internal incentive systems (Kaplan & Henderson, 2005). In other words, in the absence of factors motivating intense efforts to break free from inertia it seems likely to be the organisational status quo for some digital startups. Thus, the relatively comfortable environment of some of the participants, in which they had access to substantial training, advice, development and financial resources, together with inexpensive or free office space—may have fostered an irresistible temptation to embrace operational and strategic stagnation.

Ruef (2006) suggests that what he refers to as entrepreneurial inertia may result in an increased likelihood of boom and bust cycles, and thus potentially destabilisation of entire industries. This may be caused, for instance, by investors’ perception that industries plagued with inertia do not offer favourable returns on investment. In addition, Gong et al. (2009) propose that ‘absorptive inertia’—lack of willingness to absorb relevant information from external network sources—may be directly related to and influenced by the level of experience in an organisation. They also found that the phenomenon was linked to the perception of arrogance by some startup customers. Since some interviewees were relatively inexperienced and some level of inertia was apparent in almost all cases, it would seem disingenuous to suggest that our results mirror these results. None of the interviewees exhibited any arrogance in their interactions with the interviewer. However, lack of knowledge transfer from outside the organisation may well have contributed to inertia of the startups with more experienced managers.

### Pivoting

Ries (2011) presents pivoting as an essential component of a startup’s raison d’etre; “to turn ideas into products, measure how customers respond, and then learn whether to pivot or persevere”. While pivoting will change a startup’s strategy to pursue a vision, it rarely changes the vision. Bajwa et al. (2016), Bajwa et al. (2017), Ochoa-Zambrano and Garbajosa (2017), Bohn and Kundisch (2018), Hirvikoski (2014) and Terho et al. (2015) have collectively identified at least 14 types of pivot. There is some divergence among these authors, as Bajwa et al. (2017) identified three novel pivot types and Hirvikoski (2014) presented a social pivot type that has not been universally accepted by other authors. However, apart from these differences there is general concurrence regarding the recognized sets.

Our results suggest that digital startups hosted by the three focal incubators executed relatively diverse types of pivots, most commonly (in no particular order)—complete, customer segment, customer need, business architecture, value capture, channel, social and technology pivots. Previous studies present an unnuanced view of pivots, suggesting an almost overly romantic and simplistic view of the need for pivoting. This study offers a slightly more nuanced view. However, the most important aspect of the results was not necessarily the existence of these pivots but how the startups arrived at the decision that such a course of action was (or was not) required. It would seem reasonable to assume that startups within an incubator setting would be guided towards such an essential developmental component by stakeholders within the incubators, but that does not appear to have occurred in any meaningful way. Whether this omission was a simple oversight, due to the incubator coaches and managers not seeing this as their role, or lack of expertise and/or experience to undertake this task is not clear.

Another clearly important aspect of pivoting to consider is the environmental triggers that lead to it. As already outlined, 14 of these—11 external and three internal—have been defined by Bajwa et al. (2016) and Bajwa et al. (2017). This study identified a diverse group of externally positioned triggers comprising technology challenges, negative customer feedback and legal issues. Identified internal triggers included a flawed business model and an unscalable business. As already mentioned, a number of recent pivots were directly attributable to the impact of the novel coronavirus. It is not easy to assign this to any of the 14 pivot triggers identified in the literature, although it has undoubtedly triggered pivots. Thus, we suggest another possible category of *catastrophe-related* triggers. However, for the most part, the extant literature and findings are consistent in terms of both the existence and categorisation of pivot triggers.

### Failure/disintegration

As noted by Ries (2011), a proportion of startups will always fail/disintegrate, at least 90% according to Cusumano (2013) and Marmer et al. (2012). No serious researcher is likely to disagree, but a more debatable issue is whether failure should be seen as a net positive or net negative outcome. Mitchell et al. (2004) hypothesise that “Entrepreneurs who have failed will have higher levels of expertise relative to experience than those entrepreneurs who have not failed”. Cassar and Craig (2009) temper this enthusiasm for failure by demonstrating that entrepreneurs may tend to paint a rosier picture of the circumstances and reasons for their failure than justified by the facts, and their views may strongly depend on the failure’s nature. Important factors may include the lessons from a failure, involvement of bad luck or poor judgement, and associated constraints of the potential for future entrepreneurial efforts.

One of the key findings from this study concerns the lack of consensus regarding the meaning of failure. The interviews did not uncover any real evidence of failure/disintegration, but certainly uncovered a lack of consistency in the meaning ascribed to the terms by the participants. Moreover, the geographical setting was very limited and more variation in both the terms’ meaning and associated stigma among digital startups’ managers and general society may have been detected if the study had been extended to other regions, countries and continents.

An important debate in digital entrepreneurship research is whether opportunities are simply discovered by digital entrepreneurs or must be manufactured to avoid failure/disintegration. Shane (2012) holds that while the opportunities themselves are objective and identified by those with an entrepreneurial spirit, what is created and more subjective is the response in terms of recombination of resources by entrepreneurial organisations to exploit these opportunities. In contrast, Alvarez and Barney (2007) suggest that opportunities may be outcomes of entrepreneurial endeavours. The high level of inertia exhibited by the digital startups included in this study suggests that they may subscribe to the former view, although this possibility was not specifically addressed during the interviews.

In summary, failure/disintegration clearly occurs in lifecycles of most startups, digital or otherwise. However, it had not reportedly occurred in the startups specifically considered here. Terminology interpretation and cultural stigma reasons aside, this suggests that other factors may be at work, such as inertia. Whether inertia induces failure avoidance behaviour or vice versa is an interesting but, at this stage, unresolved issue.

### Implications for practice

This section discusses operational and strategic implications of this study that may be valuable for key stakeholders in both digital startups and incubators.

The four strategies recognised by Nair and Blomquist (2018) to minimise organisational failure/disintegration—focusing on the team around a scalable idea and business model validation, development and scalability—have already been discussed. Avoidance of failure is, of course, a desirable outcome. However, if digital startups in incubators are to evolve and reach their potential, the aspiration and focus must not be simply to elude failure, but also to pivot, scale and avoid inertia. Awareness of this need must permeate interactions of both incubators and their business coaches with digital startups. In addition, startups must overcome their apparent fear of failure/disintegration, and recognise both its likelihood and that failure will provide opportunities to try again with an enhanced skillset as well as deeper reservoirs of resilience, determination and experience.

While scaling is mentioned twice in the strategies referred to in the above paragraph, our results suggest that it warrants considerably more attention as an objective, in accordance with conclusions of Picken (2017) and Ries (2011). Picken (2017) specifically suggests that the lifecycle of an entrepreneurial venture should consist of distinct phases, such as startup, scaling and exit. However, the vast majority of digital startups do not seem to progress beyond the first phase, and even scaling remains a distant, lofty objective. Thus, incubators should focus more on paths to such growth. An eminently practical and potentially quick route to organic growth for digital startups could be the previously discussed ‘Scaling Mechanism’ involving strategic partnerships presented by Henfridsson and Bygstad (2013).

Modification of practice in line with implications of the results discussed in the previous two paragraphs should go some way to addressing problematic inertia in startups and incubators. Incubators should also strive to ensure that the comfortable environment offered to their incubatees is not taken for granted and does not result in stagnation becoming much more prevalent than pivoting and scaling activities. All parties should bear in mind potential pitfalls associated with inertia previously discussed by Ruef (2006) and Gong et al. (2009). Aggressive short- and long-term growth goals must be set, monitored, adhered to and achieved by incubators, coaches and startups synergistically and collaboratively. If these targets are not—at the very least—questions must be asked, actions taken and consequences managed.

Incubator business coaches offer guidance on operational and tactical matters and the incubators themselves offer a wide range of services, opportunities and benefits. It is right that they do so, but it must not be at the expense of more strategically focused initiatives, such as pivoting. Tremendous execution is a massive waste of time and resources if the plant is fundamentally flawed. It is, therefore, essential to raise pivoting far higher up the agenda of incubators, coaches and digital startups. Ideas must be iteratively tested, assessed and discarded until a feasible market offering is found. The famous words of Ries (2011)—“build, measure and learn”—must become the mantra for all parties in all that they do.

### Limitations

Like all investigations, this study has a number of limitations, which are briefly discussed here. First, the empirical research was restricted to three incubators in a small Swedish city, which have distinct features and sectoral foci, but also strong similarities in terms of controlling stakeholders and ownership structure. Thus, equivalent organisations in other parts of Sweden and others countries, and associated factors, may be substantially more heterogeneous. In addition, while 13 participants may be an adequate number of respondents for the purposes of this study, a larger and more diverse group of interviewees would likely yield richer and depth insights. Thus, any generalisation of the results should be very cautious. However, the correspondence of many of the findings with previous reports provides encouragement for their validity.

## Conclusions and further research

The objective in this study has been to discuss the environmental components that both facilitate and hinder the advancement of digital entrepreneurship endeavours that take place within business incubators. This resulted in the formulation of the following research question:* what are the enabling and inhibiting factors in digital startup evolution within an incubator setting?*

To address this question, a multiple-case study was executed, that involved three incubators, located within the city of Umeå in Northern Sweden. Two of the incubators—UBI and EXU—partner with organisations that operate, respectively, in the life science and creative industries. The third, BICF, has no particular industry focus. Thirteen semi structured interviews were undertaken with digital startups under the auspices of the incubators. The research efforts produced both some anticipated and unanticipated results.

One enabling factor in the former category was the finding that the digital startups claimed to both understand what a pivot is and to have undertaken the process of executing one or more of them. The range of pivot types undertaken is broad in character. An equivalent level of variety exists when one considers the triggers that lead to the requirement for pivots. The presence of incubators as important stakeholders in the evolution of digital entrepreneurship is also clearly a positive and enabling factor; although not one that is unable to benefit from adopting a more strategic view of their charges’ fortunes, ideally executed and facilitated by a team of coaches possessing a higher level of real world and relevant experience. Scaling should be both the ultimate enabling factor and goal for any fledgling digital organisation; however, other than in one case, this is not being achieved, and therefore, most of the organisations I interviewed are undoubtedly not achieving their full potential.

A number of inhibiting factors also emerged, including both surprisingly and troublingly prevalent inertia, possibly caused by scarcity of resources, incubator indifference, flawed entrepreneurial attitude or an excessively comfortable existence. In some ways failure/disintegration is a preferable outcome to one, where organisations simply tread water with no aspirations to scale their business. Failure/disintegration at least reduces further wasted investments of energy, money and our most precious resource of all; time. While failure itself is certainly an inhibiting factor—and one viewed with considerable negativity in Sweden—it also has considerable mitigating factors, not least the considerable experience and learning that it affords.

There is clearly scope for further exploration of the enabling and inhibiting factors. Improving understanding of incubators’ behavioural and support characteristics that could reduce inertia and failure/disintegration would undoubtedly enhance efficiency and potentially help all the principal stakeholders. Geographical extension of the exploration, to the entire Scandinavian region or EU for instance, could also provide valuable illumination. This could validate, refute or refine the findings and developed IDM, as well as providing opportunities to investigate possible regional variations and associated factors. Finally, fortunes of digital startups and incubators that do and do not follow the principles of the IDM warrant longer term comparison.

## Data Availability

Will be supplied from the corresponding author upon the request.

## Appendix 1: interview guide

### Background of company and key managers

1. Can you briefly describe the history of your company from its founding to the present?
2. Can you briefly describe the background of your senior management team?
3. Can you briefly explain your current strategy?
4. How long have you been part of this incubator?
5. Can you describe the major decisions that your organisation has taken during its lifespan?
6. What was the main obstacle you faced when your company started?
7. How have you overcome/attempted to overcome this?
8. What is your assessment of the firm’s current performance?
9. Have you personally and/or your organisation experienced rapid scaling at any point in your lifecycle?
10. Has your startup experienced failure or have you previously experienced a failed startup?

### Pivoting

1. Are you familiar with the definition of a pivot?
2. Can you provide a brief description of your pivot(s)?
3. At what point in your company journey did it/they take place?
4. Who participated in the decision-making activity?
5. What was/were the trigger(s) that led to your pivot?
6. Can you describe the mechanism/process by which the triggers led to the pivot?
7. How did you evaluate pivot alternatives?
8. What data/data sources did you utilise in selecting the option you proceeded with?
9. What were the pivot outcomes?
10. Did you validate the outcomes of your pivot? If so, how? What were the results?

### Incubator influence

1. Can you describe the feedback/guidance you have received from incubator business coaches and managers?
2. What specific areas of your business operations has it related to?
3. Has any of it related to pivoting?

## Abbreviations

BICF: BIC factory
EXU: EXpression Umeå
IDM: Ideation dynamics model
MVP: Minimum viable product
SISP: Swedish incubators and science parks
UBI: Umeå biotech incubator
